# Temperature Gradient Control of the Solid Oxide Fuel Cell under Variable Load

**DOI:** 10.1021/acsomega.1c01359

**Published:** 2021-10-11

**Authors:** Haibo Huo, Kui Xu, Lixiang Cui, Hao Zhang, Jingxiang Xu, Xinghong Kuang

**Affiliations:** College of Engineering Science and Technology, Shanghai Ocean University, Shanghai 201306, China

## Abstract

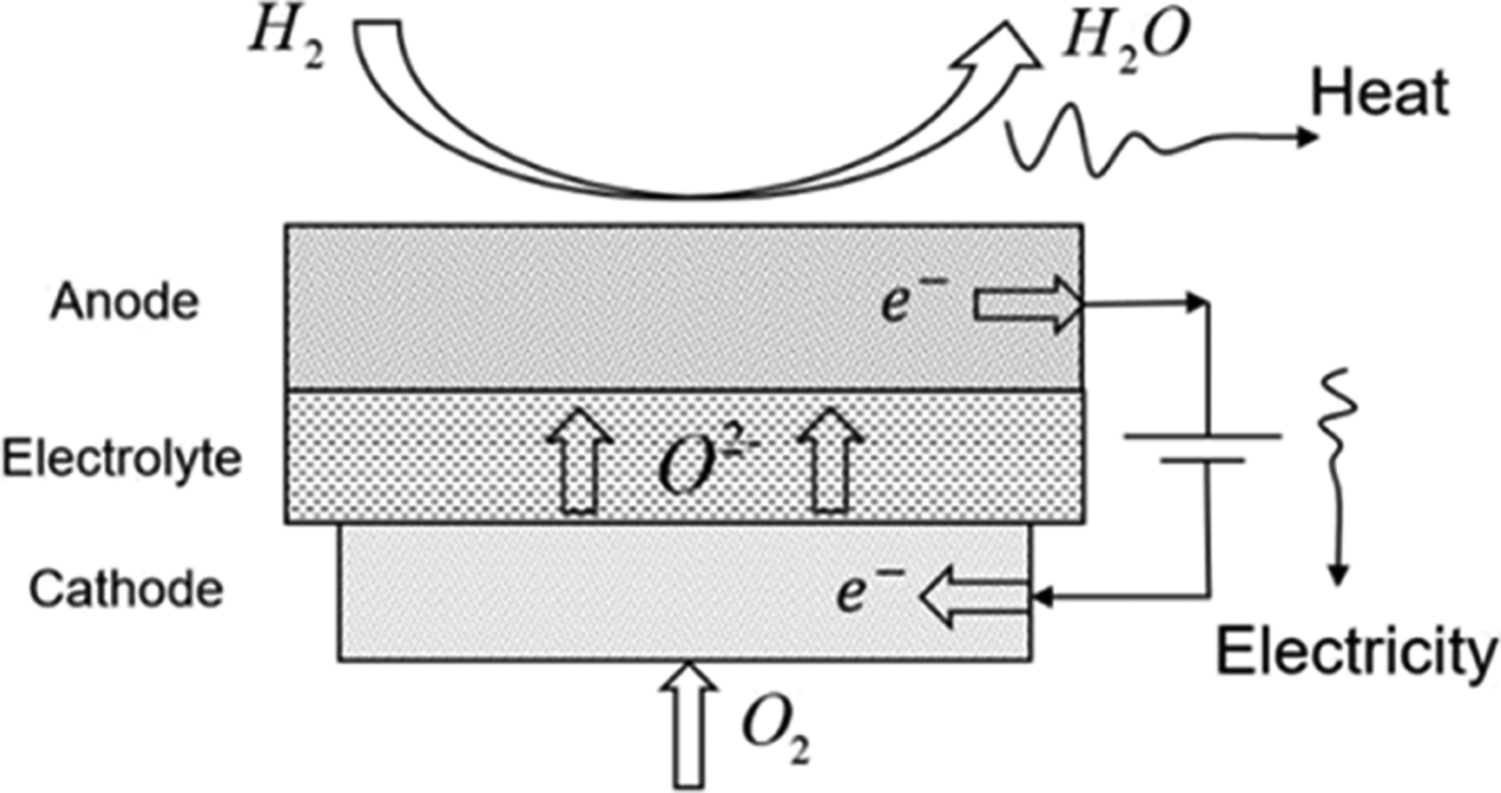

Nowadays, the temperature gradient is considered as one of the most important parameters which impact the performance of the solid oxide fuel cell (SOFC). In this paper, a control strategy based on an input–output feedback linearization technology is derived for controlling the maximum temperature gradient within the anode fuel flow channel at the desired value. For the controller design, the temperature dynamic model is proposed and simplified to a control-oriented multi-input and multioutput nonlinear dynamic model. Then, this paper presents an input–output feedback linearization controller to realize the control objective by adjusting the cathode input air flow. Finally, the simulation results are given to demonstrate the accuracy of the proposed model in reflecting the temperature dynamic characteristics. Moreover, the compound controller is added for simulation as a comparison, which shows that the proposed controller is equipped with better effectiveness and efficiency in the presence of external disturbances.

## 1 Introduction

The solid oxide fuel cell (SOFC) is an energy conversion device which directly converts chemical energy into electrical energy through an electrochemical reaction, and the chemical energy is stored in gas or gas fuel. This energy conversion jumps over the process of converting heat into mechanical energy, which brings the SOFC system not restricted by the Carnot cycle and has high energy conversion efficiency.^1^ Besides, an SOFC also has the advantages of low or zero emissions, high reliability, and no movement mechanism, making it widely usable in many fields.

The SOFC works in a complex high-temperature (600–1000 °C) environment;^2^ system nonlinearity, fuel shortage, external interference, and inaccuracy of some variables often cause a few serious problems. The temperature gradient is a key parameter of the SOFC. An excessively high temperature gradient will lead to greater thermal stress in the system, which will cause the fuel cell to deform or even crack and shorten the cell life.^3^ Therefore, to ensure the safe operation and prolong life of the SOFC, the temperature gradient needs to be controlled within a reasonable scope *T*_sol,g_^max^ ≤ 8 K·cm^–1^.^4^

By far, most of the existing controllers are for the terminal voltage or fuel utilization control of the SOFC. Several researchers have designed proportional-integral (PI) controllers for control of load current, voltage, fuel utilization, and maximum temperature of the SOFC, respectively.^5−7^ Besides, Xia et al.^8^ designed a new PID controller based on the Wiener neural network for tracking the voltage performance of the SOFC. Ławryńczuk^9^ designed a nonlinear model predictive controller to achieve satisfying voltage control. Yu et al.^10^ presented a current feedback controller combined with a static synchronous compensation technique to control the voltage.

However, there are only a few research studies on the control strategy of the temperature gradient. Kulikovsky^11^ put forward a simple equation of temperature gradient in the SOFC stack. Zeng et al.^12^ summarized the commonly used thermal management methods for temperature gradient reduction in the SOFC stack. Also, Wu et al.^13^ designed a nonlinear compound controller to control the SOFC temperature gradient. However, the detailed design process of the compound controller was not described and the control precision can also be improved.

Furthermore, most of the mechanism models of the SOFC in the above research studies have fewer coupling elements. They are very useful to analyze the dynamic performance but not suitable for designing control schemes of the complex SOFC system. To develop effective temperature gradient control strategies, a control-oriented nonlinear dynamic model of the SOFC is first proposed in this paper.

Input–output feedback linearization control is well known for its ability to generate a nonlinear control input, which yields a linear relationship between the inputs and the outputs and eventually permits the use of the conventional linear control approach.^14^ This makes the design of control law more flexible. Until now, input–output feedback linearization control has been successfully applied in many fields, such as the motor, the distributed solar collectors, friction systems, and so forth.^15−20^ However, the concrete study of input–output feedback linearization controller design for temperature gradient control of the SOFC has not been found in prior literature.

To improve durability and prevent potential failures, a nonlinear controller based on the input–output feedback linearization is proposed to control the temperature gradient within the required range in this study. The contributions of this paper are given as follows in brief:

(1) For the controller design, a control-oriented multi-input and multioutput nonlinear dynamic model, which can accurately reflect the temperature dynamic characteristics of the SOFC, is proposed.
(2) An input–output feedback linearization controller is derived for controlling the maximum temperature gradient within the anode fuel flow channel at the desired value by adjusting the cathode input air flow. Furthermore, the better effectiveness and efficiency in the presence of external disturbances of the proposed controller is demonstrated by comparing with the compound controller.

The rest of this article is arranged as follows. In Section 2, the SOFC dynamic mechanism model proposed in refs (21) and (22) is reviewed in brief, and a control-oriented nonlinear temperature gradient dynamic model is proposed. Then, the controller based on input–output feedback linearization is designed in Section 3. It is followed by Section 4, where the temperature simulation result can verify the accuracy of the dynamic model, and the maximum temperature gradient control result is depicted to show the validity of the presented controller. Finally, conclusions are summarized in Section 5.

## 2 Dynamic Model of the SOFC

### 2.1 Nonlinear Dynamic Mechanism Model

Based on the work reported in refs (21) and (22), the SOFC dynamic mechanism model is reviewed in this section.

**Figure 1:**
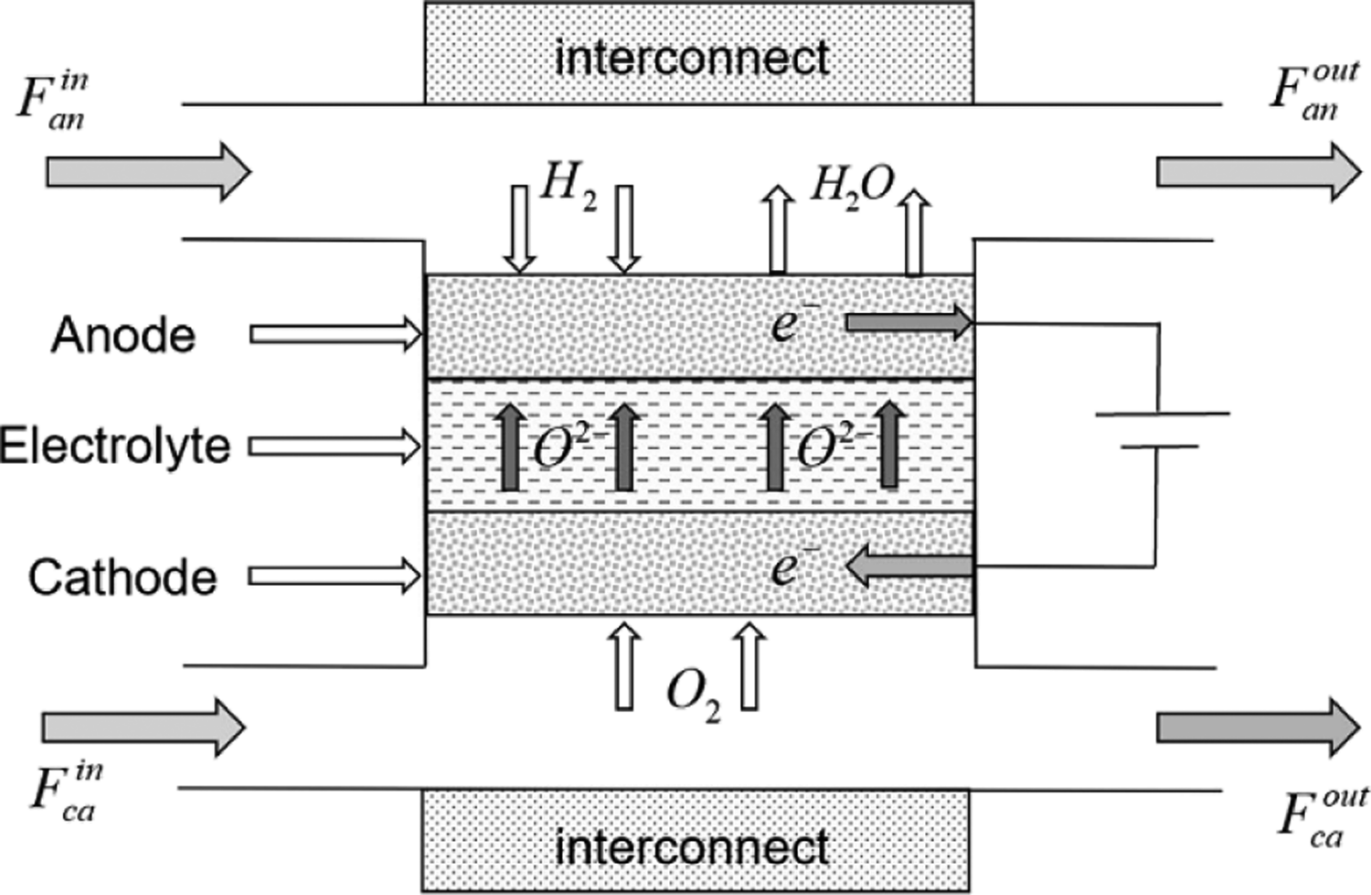
Structure diagram of the single SOFC operation.

**Figure 2:**
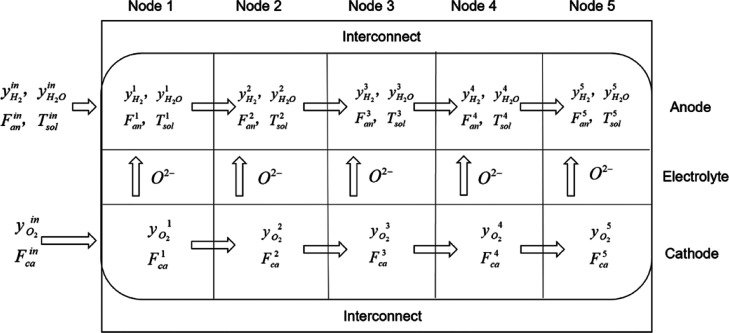
Finite volume scheme on the SOFC.

In this paper, a coflow planar SOFC stack composed of 30 single cells is studied. Figure 1 describes the basic structure and operating principle of the single cell. Assuming the reaction processes in the individual cells are the same, the SOFC stack model can be derived from the cell model. To obtain the spatial distribution of temperature and compromise the accuracy and calculation amount, the cell is divided equidistantly into five nodes along the gas flow direction from the inlet to outlet.^23,24^ The finite volume method can realize local conservation in the corresponding unit and is also suitable for dealing with complex range and boundary problems.^25^ Therefore, in this study, the finite volume method is utilized to build the model for the single cell, as shown in Figure 2.

For each mode, the lumped parameter dynamic model includes three parts, that is, mass balance submodel, thermal balance submodel, and electrochemical submodel.

#### 2.1.1 Mass Balance Submodel

Equations 1 and 2 give the mass balance models in the cathode channel, which provide the mole fraction of oxygen in the SOFC. , , and , respectively, represent the mole fractions of oxygen, hydrogen, and water vapor in the *m*-th (*m* = 1, 2, 3, 4, 5) node.
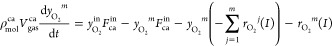
1
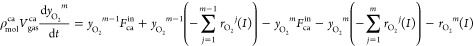
2where, *m* = 1 for eq 1 and *m* = 2, 3, 4, 5 for eq 2, ρ_mol_^ca^ and *V*_gas_^ca^, respectively, represent the molar density and volume in the cathode channel,  is regarded as the reaction rate of oxygen, *F*_ca_^in^ means the cathode input air flow, and the anode input fuel flow *F*_an_^in^ is described as follows
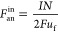
3where *I* is the stack current, *N* represents the number of cells in the stack, *u*_f_ means the fuel utilization, and *F* denotes the Faraday constant.

Similarly, the mass balance submodels of hydrogen and water vapor in the anode fuel channel are given as follows
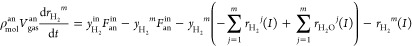
4
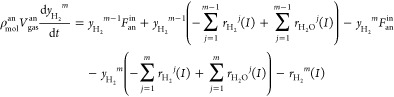
5
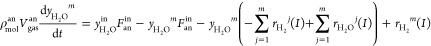
6
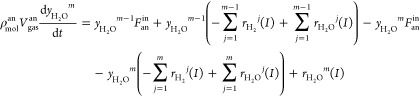
7where *m* = 1 for eqs 4 and 6, and *m* = 2, 3, 4, 5 for eqs 5 and 7, ρ_mol_^an^ represents the anode molar density, *V*_gas_^an^ is the anode gas channel volume, and  and  are the reaction rates of hydrogen and water vapor, respectively.

In the above formulas, the reaction rates are
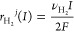
8
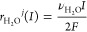
9
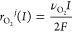
10where *v* represents the stoichiometric coefficient.

#### 2.1.2 Thermal Balance Submodel

The temperature of the solid structure is one of the most important operating variables that affects the performance of the SOFC because the heat-transfer coefficients of the solid structure, namely, of the PEN and the interconnector, are much smaller than those of the gases. Thus, the PEN and interconnector temperatures are assumed to be equal. Since the anode fuel flow rate *F*_an_^in^ = *IN*/2*Fu*_f_ (about 2 × 10^–4^ mol s^–1^) is much smaller than the cathode air flow rate (2.742 × 10^–3^ mol s^–1^),^21^ the fuel has enough time for chemical reaction and heat exchange with the solid structure. In this paper, we assume that the anode fuel temperature equals that of the solid structure in each node. The thermal balance equations for the solid structure are described as
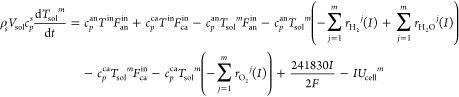
11
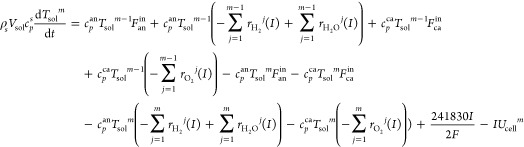
12where, *m* = 1 for eq 11 and *m* = 2, 3, 4, 5 for eq 12, *V*_sol_ is the volume of the solid structure, *c*_*p*_^an^ and *c*_*p*_^ca^ mean the specific heat capacity in the anode and the cathode, respectively, *T*^in^ represents the anode input fuel temperature, and 241830*I*/2*F* is the energy density.

#### 2.1.3 Electrochemical Submodel

Considering the effect of the ohmic losses and the polarization losses, the voltage in each node of the SOFC can be represented by
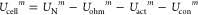
13where *E*_*N*_^*m*^ is the open circuit voltage of the *m*-th node, *U*_ohm_^*m*^, *U*_act_^*m*^, and *U*_con_^*m*^, respectively, represent the ohmic losses, the activation losses, and the concentration losses of the *m*-th node.
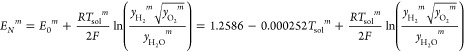
14
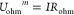
15
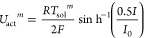
16
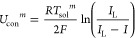
17where *I*_L_ and *I*_0_ denote the limited current and the change current, respectively. *R* is the universal gas constant, and 1.2586–0.000252*T*_sol_^*m*^ represents the standard cell potential, which is *E*_0_^*m*^ in eq 14.

In fact, in a SOFC operation, the flow, thermal, chemical, and electrochemical (EC) processes are intrinsically coupled. Heat generation and absorption by the chemical and EC reactions in turn affect the temperature distribution and gas flow composition.

## 3 Control-Oriented Nonlinear Temperature Gradient Model

To design suitable control strategies for temperature gradient control, a control-oriented nonlinear dynamic model needs to be established based on the SOFC thermal balance submodel. For the controller design, the node temperatures are taken as the state variables, namely, *x* = [*x*_1_, *x*_2_, *x*_3_, *x*_4_, *x*_5_]^*T*^ = [*T*_sol_^1^, *T*_sol_^2^, *T*_sol_^3^, *T*_sol_^4^, *T*_sol_^5^]^*T*^, the output variable is the maximum temperature gradient among the five nodes, that is, ***y*** = *T*_sol,*g*_^max^, which is within the anode fuel flow channel, the current is regarded as a bounded disturbance, that is, Δ*j* = *I*, and the cathode input air flow is chosen as the manipulated variable. According to the temperature dynamics, as shown in eqs 11 and 12, the nonlinear dynamic model of the SOFC can be built as
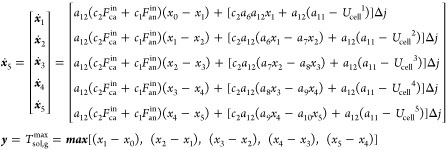
18where, *x*_0_ means the initial temperature of the solid structure, which is also the anode input fuel temperature. Furthermore, several notations define the parameters below
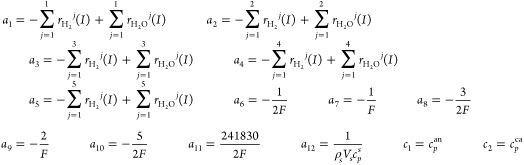


In this paper, we control the temperature gradient by adjusting the cathode input air flow. To design the input–output feedback linearization controller, according to eq 18, the control-oriented nonlinear temperature gradient model can be derived as
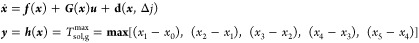
19where the control input ***u*** is the cathode input air flow, and
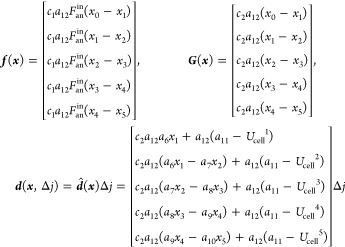
20

## 4 Temperature Gradient Control of the SOFC

### 4.1 Input–Output Feedback Linearization

In recent years, feedback linearization has become a popular nonlinear system control method, which is widely used in the decoupling, output regulation and response tracking of nonlinear systems. Feedback linearization is conveniently classified into categories, that is, input–output feedback linearization and state-space linearization. Input–output feedback linearization technology provides convenience for further design of linearization control algorithm by transforming the nonlinear system model (in whole or part) into a linear system.^26,27^ The standard input and output feedback linearization technology is described as follows.

Consider the following nonlinear dynamic model
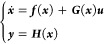
21where ***x*** ∈ ***R***^*n*^  is the state vector, ***u*** ∈ ***R***^*m*^ is the control input, ***y*** ∈ ***R***^*m*^ is the system output, ***f***(***x***), ***G***(***x***) = [*g*_1_(***x***),..., *g*_*m*_(***x***)], ***H***(***x***) = [*h*_1_(***x***),..., *h*_*m*_(***x***)]^*T*^ and *g*_*i*_ (*i* = 1,..., *m*) are all *n* dimensions smooth vector fields, and *h*_*i*_ (*i* = 1,..., *m*) is the sufficiently smooth scalar function.

We define the first derivative of the output variable ***y*** as follows
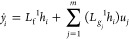
22where *L*_f_^1^*h*_*i*_ = ∂*h*_*i*_(***x***)/∂***xf***(***x***), *L*_g*j*_^1^*h*_*i*_ = ∂*h*_*i*_(***x***)/∂***x****g*_*i*_(***x***).

If , the first derivative of ***y*** is connected to the control law ***u***. The control law can be directly derived as ,^28,29^ where *v* denotes the derivative of ***y*** and can obtain linear relationships between the inputs and outputs.^14^ Supposing that all the , it means that the derivative of ***y*** has no relationship with the control law ***u***; then, the derivation needs to be continued. Let us assume that λ_*j*_ is the minimum integer and at least one  not to be zero, that is
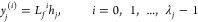
23
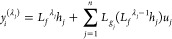
24

Thus, the control law ***u*** is
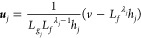
25

### 4.2 Model Linearization and Controller Design

When a load disturbance causes the current change, the control objective is to exactly track the desired maximum temperature gradient by controlling the cathode input air flow. Applying the input–output feedback linearization technique described in Section 4.1, the first-time derivative of ***y*** in eq 19 can be derived as
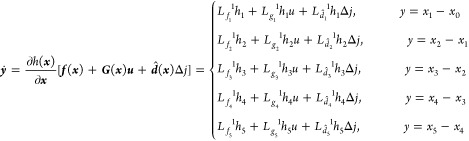
26where
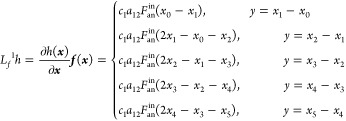
27
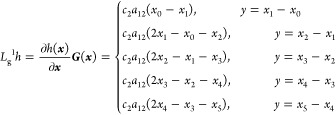
28
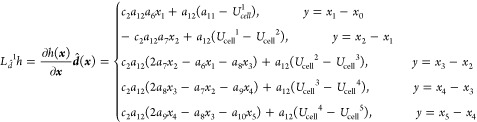
29

Due to all the , the first derivative of the output variable ***y*** has a direct relationship with the control input ***u***. Thus, the control law can be described as
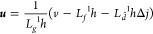
30here, we define a track error *e* = *y*_d_ – *y*, where *y*_*d*_ is the desired value of the maximum temperature gradient. However, in a nonlinear dynamic system, even though it can be accurately linearized, tracking errors may exist as the parameters change, especially in the case where the load changes. Therefore, to eliminate the error, a new control input of the following form is introduced
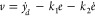
31where *v* is the first-time derivative of *y*, that is, *ẏ* = *v*; then, we have
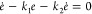
32

By tuning the parameters *k*_1_ and *k*_2_, the characteristic polynomial of 32 is the Hurwitz polynomial; this means that the tracking error *e* converges to zero and the established controller is effective.^28^

## 5 Results and Discussion

In the MATLAB/Simulink environment, the validation of the SOFC dynamic model is first verified. Then, the input–output feedback linear control strategy is simulated and the corresponding temperature gradient control results are described in this section.

### 5.1 Model Verification

In this paper, we choose the cathode input air flow and the anode input fuel flow as the model inputs, and the five nodes’ temperatures of the SOFC as the outputs. The parameter setting values used in eqs 1–17 are given in Table 1. The initial conditions are given as follows: the input fuel temperature is *T*_in_ = 1000 K, the initial temperature of the air in the cathode flow channel is 1060 K, the cathode input air flow rate is *F*_ca_^in^ = 2.742 × 10^–3^ mol ·s^–1^,^21^ and the current value is set as 2A. To ensure uniform distribution of the temperature and the voltage inside the SOFC, the fuel utilization is chosen as *u*_f_ = 0.85.^30^

**Table 1:** Modeling Parameters of the SOFC

symbol	definition	value (unit)
*c*_*p*_^an^	anode specific heat capacity	27.3032 J·mol^–1^·K^–1^
*c*_*p*_^ca^	cathode specific heat capacity	35.0058 J·mol^–1^·K^–1^
ρ_mol_^an^	anode molar density	32222 mol·m^–3^
ρ_mol_^ca^	cathode molar density	22839 mol·m^–3^
*V*_gas_^an^	anode channel volume	1.62 × 10^–6^ m^3^
*V*_gas_^ca^	cathode channel volume	3.24 × 10^–6^*m*^3^
*I*_L_	limit current	100 A
*I*_0_	exchange current	80 A
*R*_ohm_	ohm resistance	200 Ω
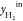	the initial mole fraction of hydrogen	0.97
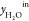	the initial mole fraction of water vapor	0.03
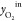	the initial mole fraction of oxygen	0.21
*F*	Faraday constant	96485 C·m ol^–1^
*R*	ideal gas constant	8.3142 J·mol^–1^·K^–1^
*T*_in_	input fuel temperature	1000 K
*N*	number of cells	20

**Figure 3:**
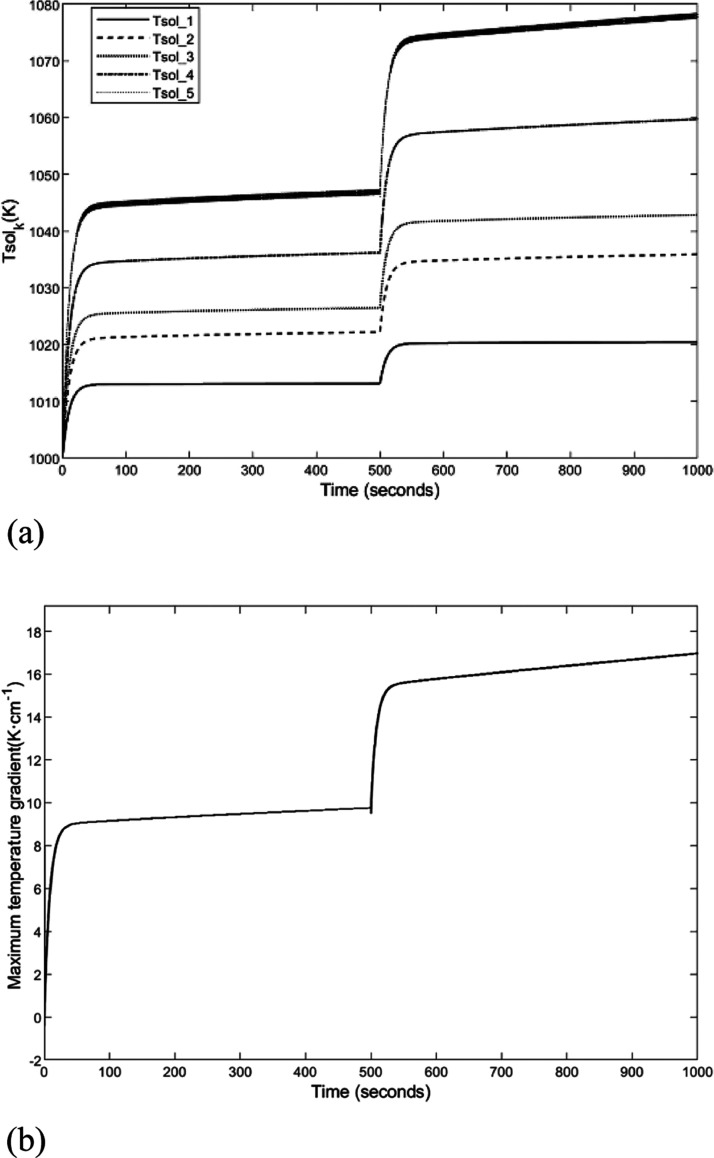
Simulation diagram of the temperature dynamic characteristics. (a) Five-node temperature dynamic response curve. (b) Maximum temperature gradient dynamic response curve.

During the normal operation of the SOFC, a load disturbance causes the stack current to have a step change (from 2A to 3A) at *t* = 500 s. In this situation, the five nodes’ temperature dynamic characteristic curves and maximum temperature gradient response graphic are depicted as Figure 3. According to the temperature distribution, the node temperatures have a smooth increase when there is a step increase in the current. This is because the load current increasing will cause more intensive electrochemical reaction. Thus, it will release more heat and gradually increase the stack temperature in each node. As the temperature changes, the maximum temperature gradient also changes. It can be seen from Figure 3a that the temperature of the five nodes satisfies *T*_sol_^1^ < *T*_sol_^2^ < *T*_sol_^3^ < *T*_sol_^4^ < *T*_sol_^5^. Furthermore, the maximum temperature gradient of the increasing trend can be seen in Figure 3b, which is the same as the objective situation. These descriptions indicate that the dynamic model presented in this paper can be applied to accurately describe the dynamic characteristics of the SOFC.

### 5.2 Temperature Gradient Control

In general, as the load current increases, the SOFC stack temperature rises because the molar concentration of reactants along the direction of the gas flow gradually decreases. Due to larger cathode input air flow and the specific heat capacity of the air is higher than that of the fuel, the stack temperature gradient can be reduced by increasing the cathode input air flow. In this study, the maximum temperature gradient within the anode fuel flow channel of the SOFC is controlled by adjusting the cathode input air flow.

**Figure 4:**
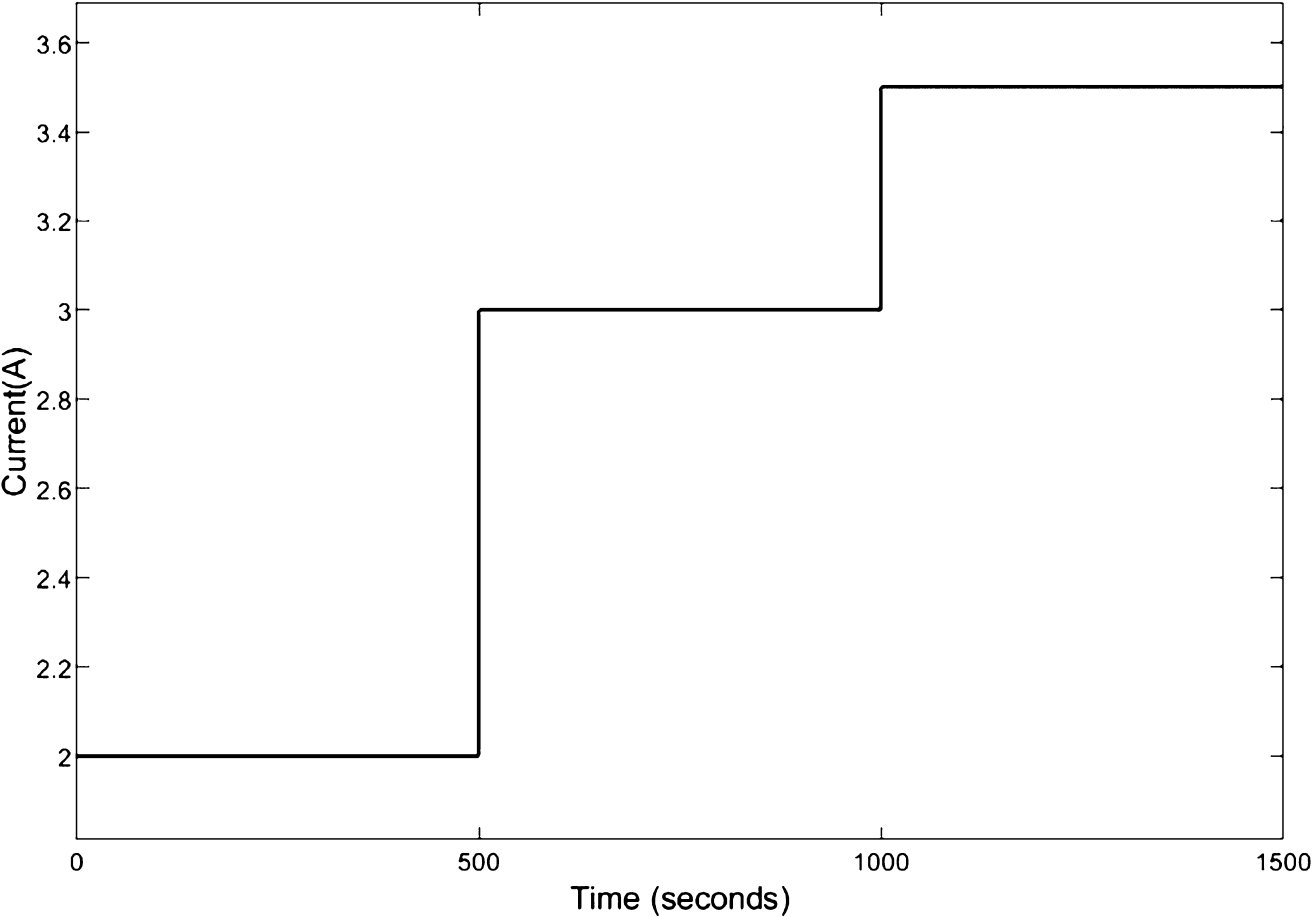
Step curve of current.

**Figure 5:**
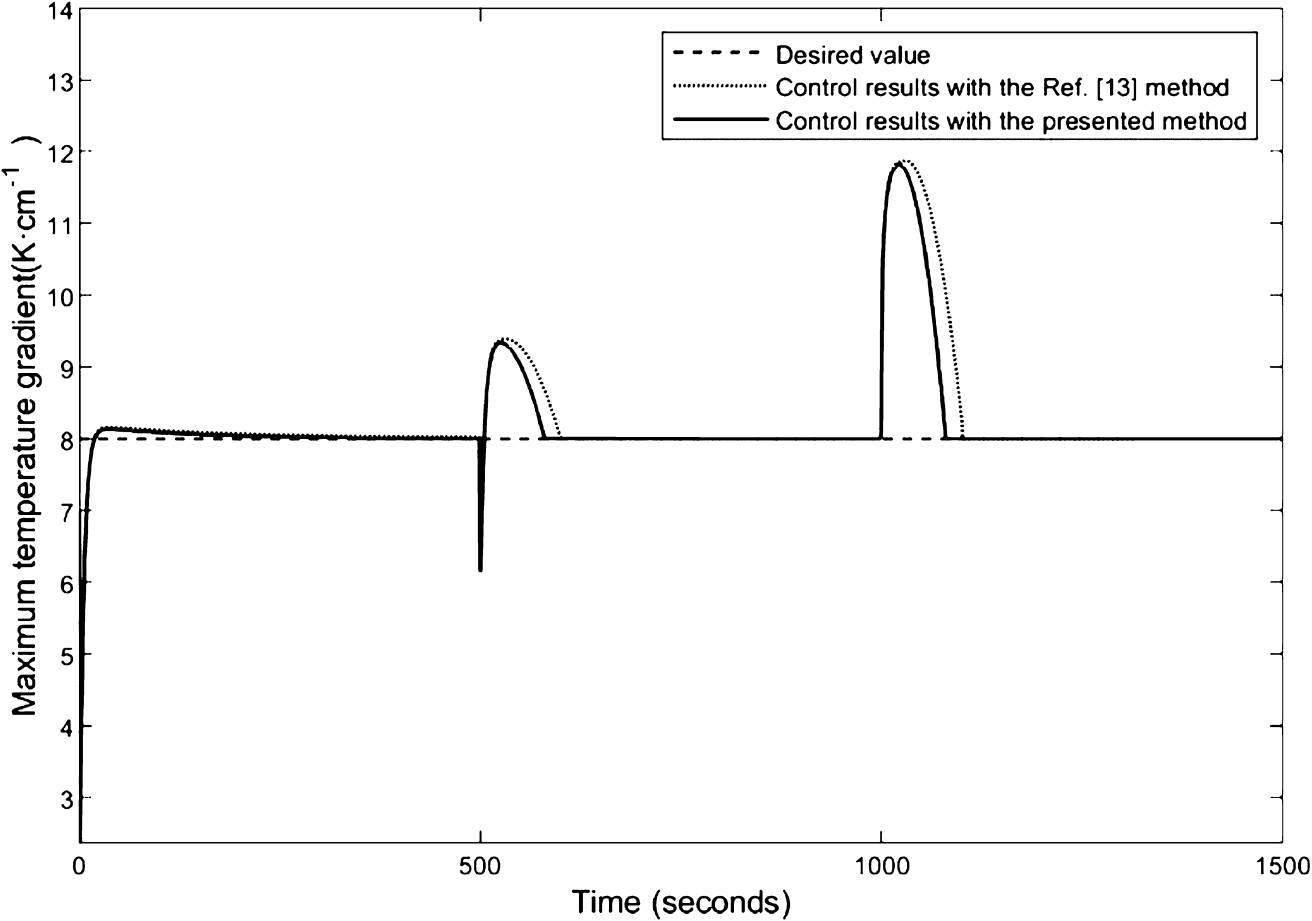
Maximum temperature gradient control results of the SOFC using the presented controller and the method in ref (13).

To access the control performance of the proposed control strategy, we choose the current disturbance as a multiple-step signal which increases from 2 to 3 A at 500 s and goes on to 3.5 A after 1000 s. The current change curve is shown in Figure 4. Based on the control-oriented model of the SOFC, the input–output feedback linearization control strategy can be developed. In this study, the control objective is to maintain the maximum temperature gradient of the SOFC as the desired value (*T*_sol,g,ref_^ma*x*^ = 8 K·cm^–1^) by adjusting the cathode input air flow. Using the input–output feedback linearization control strategy, combining pole placement technique, where the parameters are tuned as *k*_1_ = 25 and *k*_2_ = 1.25, the presented temperature gradient control scheme is simulated and the result is described by a solid line. For the purpose of comparison, the compound control law proposed in ref (13) is also used to control the maximum temperature gradient and the response is depicted by a dashed line; their comparison results are shown in Figure 5.

From Figure 5, one will notice that both the above controllers can ensure the maximum temperature gradient of the SOFC to the desired value accurately. In the case of the above disturbances, it is worth to note that the maximum temperature gradient response rate for the control method in the ref (13) is much slower than that for the presented controller under the same control objective. This clearly proves the favorable performance of the presented controller for the maximum temperature gradient control of the SOFC.

**Figure 6:**
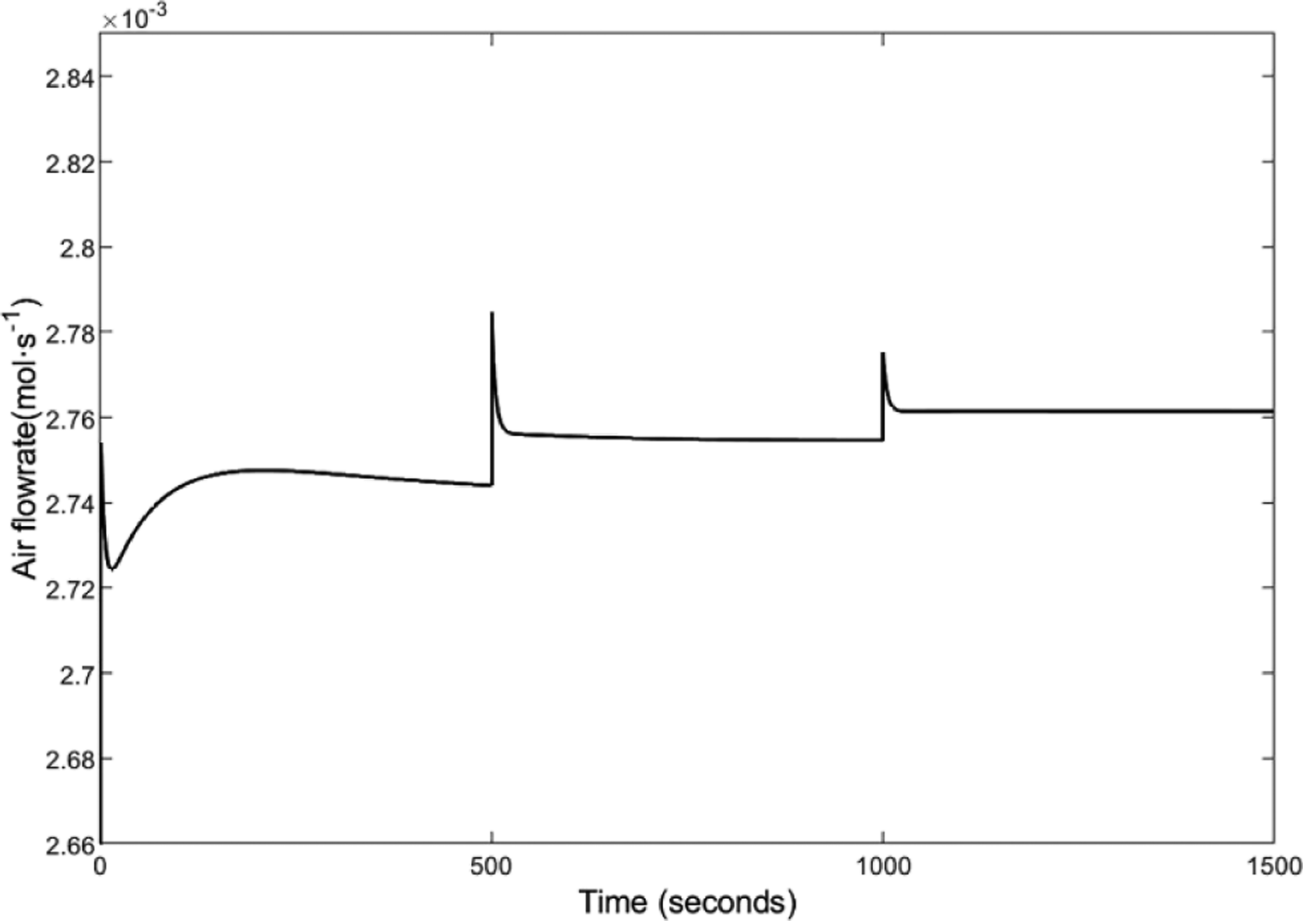
Curve of the control input (the cathode input air flow).

To eliminate the real-time error between the actual and the desired maximum temperature gradient, the control input (the cathode input air flow) is given as Figure 6. As can be seen from Figure 6, when the current increases, the maximum temperature gradient of the SOFC can stabilize at the desired value by increasing the cathode input air flow.

## 6 Conclusions

The main task of this study is to control the maximum temperature gradient at its desired value in the presence of the load current. To satisfy the variable requirements of the control strategy, a control-oriented temperature-gradient nonlinear dynamic model of the SOFC is first established. Simulations show the feasibility of the established model, which can accurately reflect the steady state and transient operation of the temperature at each node.

Then, an input–output feedback linearization controller for the maximum temperature gradient control is proposed to make the control objective come true. The result shows that the proposed controller has the better control performance by comparing with the compound controller. Although the maximum temperature gradient can reach the same steady-state value under both control laws, the presented controller possesses the characteristics of faster response time and higher control precision.

As the future work. we ought to focus on the dynamic characteristics of the air temperature and its effect on the temperature gradient of the solid structure of the SOFC, rather than simply assuming that the temperatures of the fuel and the solid structure are the same. Furthermore, applying the presented temperature gradient controller to a more complicated and truer SOFC system proves the theoretical feasibility.

## Supporting Information Available

Calculation equations of each data in Table 1 and its corresponding initial data (PDF)

## Nomenclature

*F*: mole flow rate (mol·s^–1^) or Faraday’s constants: (96485 C·m ol^–1^)
*V*: volume (m^3^)
*y*: mole fraction
*r*: reaction rate term
*I*: stack current (*A*)
*I*_0_: change current (*A*)
*I*_L_: limited current (*A*)
*N*: numbers of cells in the stack
*R*: universal gas constant (8.314 J·mol^–1^·K^–1^)
*R*_ohm_: ohmic resistance (Ω)
*c*_p_: specific heat capacity (J·mol^–1^·K^–1^)
*u*_f_: fuel utilization
*T*: temperature (*K*)
*U*: voltage (*V*)
*E*_N_: open-circuit voltage (*V*)
