# Supplementary material for: Temperature Gradient Control of the Solid Oxide Fuel Cell under Variable Load
Source: ACS Omega. 2021 Oct 11;6(42):27610–9. doi: 10.1021/acsomega.1c01359 (PMC8552236; doi:10.1021/acsomega.1c01359)
Supplement: Supplementary file 1 — ao1c01359_si_001.pdf [file ao1c01359_si_001.pdf]

Supporting Information

Temperature Gradient Control of Solid Oxide Fuel Cell Under Variable Load

Haibo Huo*, Kui Xu, Lixiang Cui, Hao Zhang, Jingxiang Xu, and Xinghong Kuang**

College of Engineering Science and Technology, Shanghai Ocean University, Shanghai 201306, China

*E-mail: hbhuo@shou.edu.cn (H.B. Huo)

**E-mail: xhkuang@shou.edu.cn (X.H. Kuang)

Some of the parameters in Table. 1 of the manuscript give the following formula:

$$c_p^{an} = y_{H_2}^{in} \times c_p^{H_2} + y_{H_2O}^{in} \times c_p^{H_2O} \tag{S1}$$

$$c_p^{ca} = y_{O_2}^{in} \times c_p^{O_2} + y_{N_2}^{in} \times c_p^{N_2} \tag{S2}$$

$$A_{node} = W_{cell} \times (\frac{L_{cell}}{5}) \tag{S3}$$

$$A_{gas} = A_{node} \times P_{active} \tag{S4}$$

$$V_{gas}^{a} = H_a \times A_{gas} \tag{S5}$$

$$V_{gas}^{c} = H_c \times A_{gas} \tag{S6}$$

Table S1. Parameters in the above formula.

Symbol	Definition	Value (Unit)
$c_p^{H_2}$	Specific heat capacity of hydrogen	$27.2342\ J/(mol \cdot K)$
$c_p^{H_2O}$	Specific heat capacity of water vapor	$29.5086\ J/(mol \cdot K)$
$c_p^{O_2}$	Specific heat capacity of oxygen	$27.2353\ J/(mol \cdot K)$
$c_p^{N_2}$	Specific heat capacity of nitrogen	$37.0714\ J/(mol \cdot K)$
n_{an}^{in}	The amount of substance of the anode inlet gas	$0.0522\ mol$
n_{ca}^{in}	The amount of substance of the cathode inlet gas	$0.074\ mol$
L_{cell}	Cell length	$0.1\ m$
W_{cell}	Cell width	$0.1\ m$
H_{an}	Height of anode channel	$0.001\ m$
H_{ca}	Height of cathode channel	$0.002\ m$
P_{active}	Proportion of active area to cell area	0.81
